# Analysing false‐positive errors when Australian radiographers use preliminary image evaluation

**DOI:** 10.1002/jmrs.809

**Published:** 2024-06-25

**Authors:** Jermayne Takapautolo, Michael Neep, Deborah Starkey

**Affiliations:** ^1^ Department of Medical Imaging Logan Hospital Meadowbrook Queensland Australia; ^2^ School of Clinical Sciences Queensland University of Technology Brisbane Australia

**Keywords:** Clinical audit, diagnostic errors, emergency department, radiographer, X‐ray

## Abstract

**Introduction:**

Diagnostic errors in the emergency departments can have major implications on patient outcomes. Preliminary Image Evaluation (PIE) is a brief comment written by a radiographer describing an acute or traumatic pathology on a radiograph and can be used to complement referrer's image interpretation in the absence of the radiologist report. Currently, no studies exist that focus their analysis on false‐positive (FP) errors in PIE. The purpose of this study was to investigate the regions of the body that cause the most FP errors and recognise other areas in image interpretation that may need additional attention.

**Methods:**

A longitudinal retrospective clinical audit was conducted to determine the accuracy of radiographer PIE's over 5 years from January 2016 to December 2020. PIE's were compared to the radiologist report to assess for diagnostic accuracy. FP and unsure errors were further categorised by anatomical region and age.

**Results:**

Over this period, a sample size of 11,090 PIE audits were included in the study demonstrating an overall PIE accuracy of 87.7%. Foot, ankle and chest regions caused the most FP errors, while ankle, shoulder and elbow caused the most unsure cases. 76% of the unsure cases were negative for any pathology when compared to the radiologist report. The paediatric population accounted for 21.3% of FP cases and 33.6% of unsure cases.

**Conclusion:**

Findings in this study should be used to tailor education specific to radiographer image interpretation. Improving radiography image interpretation skills can assist in improving referrer diagnostic accuracy, thus improving patient outcomes.

## Graphical abstract

The purpose of this study was to investigate the regions of the body that cause the most false‐positive errors. A longitudinal retrospective clinical audit was conducted to determine the accuracy of radiographer preliminary image evaluations over five years from January 2016 to December 2020. Findings in this study should be used to tailor education specific to radiographer image interpretation.

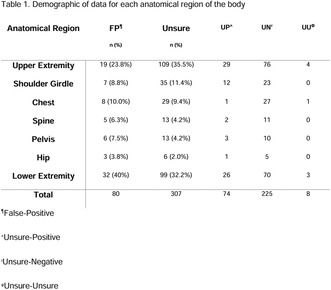

## Introduction

Diagnostic errors in the emergency department can have major implications on patient outcomes.^1^ Regardless of whether these diagnostic errors are false‐negative or false‐positive, they can still result in incorrect or suboptimal patient care. A false‐negative diagnostic error is when an acute abnormality is present, but it gets missed by the practitioner. A false‐positive error is when an acute abnormality is incorrectly identified, on a normal patient presentation. The evaluation of radiographic images by radiographers has been practiced in the United Kingdom for several decades, evolving to meet the needs of the imaging departments they serve in.^2^ Preliminary Image Evaluation (PIE) is a brief comment written by a radiographer describing an acute or traumatic pathology on a radiograph. It is important to note that PIE is not a substitute for a radiology report.^3^ A study by Brown et al.^4^ showed that radiographers were able to demonstrate a reasonably high diagnostic accuracy when using PIE. With a high diagnostic accuracy, PIE can be used to complement referrers’ image interpretation in the absence of the radiologist report. This would improve the accuracy of the diagnosis thus improving the management and outcome for patients.

Brown et al. showed that when radiographers used PIE in emergency departments, the most common errors were false‐negative (FN) errors in the upper and lower extremities.^4^ They suggested that education should be tailored to these anatomical regions.^4^ A recent study by Alexander‐Bates et al. further investigated the common FN PIE errors in the hopes to lead more content‐focused education for radiographers.^5^ The study discovered that phalanges of the hand and feet accounted for the most interpretive errors from radiographers. More recently, Petts et al.^6^ compared and combined the accuracy of radiographer's X‐ray interpretation with emergency clinicians. While the overall accuracy across both groups were similar, the types of errors were different, highlighting the importance of a having the radiographer's PIE to aid an emergency clinicians X‐ray interpretation. This was evident as combining the interpretations of both disciplines elicited a higher accuracy, sensitivity and specificity.

Although research has highlighted the benefits of PIE, there are still areas within the field that are yet to be investigated. Currently no studies exist that involve the analysis of false‐positive (FP) errors in PIE. Although it is an essential skill for a radiographer to identify abnormalities, it is equally just as important to exclude abnormalities.^7^ Generally, image interpretation training places a large emphasis of its content on identifying abnormalities despite a vast majority of patient presentations in a clinical setting being normal. The imbalance in prevalence in disease and the emphasis on abnormal presentations can impact one's practice when making interpretations.^8^ FP interpretation errors are cases that the radiographer deemed to be abnormal, but when further reviewed by the radiologist, there was no abnormality present. Although these errors only account for approximately 2% of data within Brown et al.'s research, there are still major implications that these errors can have on the patient and health system that need to be further investigated. One of the biggest issues that can come from false‐positive errors is overtreatment. Overtreatment refers to the use of an intervention that does not provide enough patient benefit to outweigh the risk of the intervention.^9^ Overtreatment is a complex and multifactorial issue that can lead to several consequences, mainly the unnecessary allocation of resources. Furthermore, there has been no investigation comparing PIE data between paediatric and adult populations. It has been well documented that paediatric image interpretation has been an area of difficulty for practitioners.^10^ Normal variants in adults also pose interpretation difficulties. In both paediatric and adult populations, it would be of interest to investigate what type of injuries caused these false‐positive errors and why they may have occurred. Besides studies by Brown and Alexander‐Bates^4^, ^5^, there is minimal research involving large sample sizes of data. The purpose of this study was to investigate the regions of the body that cause the most false‐positive errors and recognise other areas in image interpretation that may need additional attention.

## Method

### Ethics

Ethical Approval was granted from the Metro South Health Research Ethics Committee and Queensland University of Technology Human Research Ethics Committee.

### Design

A longitudinal retrospective clinical audit was conducted to determine the accuracy of radiographer PIE's over a 5‐year period from January 2016 to December 2020.

### Study setting and participants

The study was performed in the emergency department of Logan Hospital medical imaging. Logan Hospital had 455 beds and is located in South East Queensland. Over the course of the study, 11,090 PIE's were audited by 37 rotating full‐time radiographers of varying experience. PIE's were performed 24 h 7 days a week. Prior to the commencement of the study, it was mandatory that all radiographers had undergone an image interpretation training programme that included 17 modules covering the appendicular and axial skeleton, chest and abdomen. Specifically, pathologies involving bony fractures, joint disruptions, lipohaemarthrosis, foreign bodies, pneumothorax and pneumoperitoneum. Pathologies that did not meet these were considered outside the scope of PIE. While radiography students performed PIE's, their submission was always reviewed and verified under a supervising radiographer. For this reason, students were not included as participants and image interpretation training modules were not mandatory.

### Sample size calculation

A systematic method was conducted by the research team to calculate the sample size of PIE examinations that were needed to be assessed. The objective was to obtain a sample size that represented a variety of radiographic examinations, patient presentations, at various times of the day and by a wide range of radiographers. The research team concluded that all radiographic examinations within the scope of PIE for 2 days a week were going to be included. To minimise the sampling bias differing in daily patient presentations, 1 day throughout the week (Monday to Friday) and one on the weekend (Saturday and Sunday) were randomly selected using a random number generator where days corresponded to numbers 1–7. In the following week, a new weekday and weekend was randomly selected to gather a new sample of PIE's.

### Procedure

Once the patient imaging series was completed, the interpretation of the radiograph was performed on the workstation monitor by the participating radiographer or radiographers. While these monitors were of a lower resolution than one that a radiologist would use, radiographers did have access to a high‐resolution monitor if the radiograph needed further review. While there was not a time limit set to interpret the radiographs, they were still performed in a timely manner. When a traumatic or acute abnormality was present on a radiograph, the radiographer was required to annotate an ALERT on the corresponding images that demonstrated the abnormality, followed by documenting a PIE that described the pathology. The abnormality's type, site and side was recorded and later compared to the radiologist report to assess for diagnostic accuracy. Depending on the accuracy or nature of the radiographer's PIE, they were categorised into one of the following.

- True‐positive (TP)
- True‐negative (TN)
- False‐positive (FP)
- False‐negative (FN)
- False‐negative/True‐positive (FN/TP)
- True‐positive/False‐positive (TP/FP)
- Unsure
- No participation

A TP indicated that the patient had an abnormality and it was correctly identified by the radiographer. A TN indicated that the patient did not have an abnormality, and it was not identified by the radiographer. A FP meant that the radiographer incorrectly identified there was an abnormality when there was none. A FN indicated that there was an abnormality present, but it was not identified by the radiographer. A FN/TP included cases where two or more pathologies were present, but only one was identified. A TP/FP involved cases where the radiographer identified two or more pathologies, but only one was correct. Even though FN/TP and FP/TP cases include the correct identification of an acute abnormality, the PIE is still inaccurate. Since an inaccurate PIE can still negatively influence the accuracy of a diagnosis, FN/TP and FP/TP were considered errors. If the radiographer was unsure about a case, it was labelled as ‘unsure’. If the radiographer did not leave a PIE, the case was categorised as ‘no participation’. For this study, only the data from the false positive and ‘unsure’ categories were included in the detailed analysis. Unsure data was further categorised into unsure‐positive (UP), unsure‐negative (UN) and unsure‐unsure (UU). UP cases represented X‐rays where the radiographer was unable to make a definitive PIE but had an abnormality present when reviewed by the radiologist. UN cases represented X‐rays where the radiographer was unable to make a definitive PIE and did not have any abnormality. UU were cases that were inconclusive for both the radiographer and the radiologist.

### Data analysis

After PIE's were collected and sorted into their relevant diagnostic categories, descriptive statistics were used to note and describe participants’ demographic information. Cases were organised into their relevant category when compared to the radiology report. Once false‐positive and unsure cases were finalised, data including patient age and sex were retrospectively collected. Collecting patient age was of interest as there is yet to be any research involving large data sets that assess paediatric image interpretation by radiographers. Patients that were 16 years old and younger were categorised as paediatric. Furthermore, fractions of TP, TN, FP and FN were performed to calculate overall accuracy, specificity, sensitivity, positive predictive value, negative predictive value and service accuracy. Service accuracy represented the radiographer's accuracy of their PIE and is calculated by subtracting the percentage of cases where the radiographer did not participate or were unsure from the overall accuracy. This statistic was included as the authors believed it to be a more accurate representation of PIE's accuracy.

Prior to analyses, to demonstrate reliability of the audit methodology, inter‐rater and intra‐rater reliability were calculated on a subset of the sample. This involved an additional auditor and the original auditors re‐marking 5% of the total sample (*n* = 554). Favourable inter‐rater reliability (kappa <0.85 for all cases) and intra‐rater reliability (kappa <0.90 for all cases) was observed, indicating a reliable audit process.

## Results

Over the 5 year study period, a sample size of 11,090 PIE audits were included in the study. The overall accuracy, sensitivity and specificity of the radiographers PIE's were 87.7%, 79.1% and 95.8%, respectively. Table 1 displays a summary of the anatomical regions and the relevant category that region was allocated to when compared to the radiologist report. A further summary for anatomical regions within upper and lower extremities can be seen in Table 2. Of the sample, there were 80 FP cases (Table 3). This represented 0.7% of the sample. As demonstrated in Table 4, the three most common regions that lead to a FP error were the foot, ankle and chest with 13, 10 and 8 cases, respectively. Of the FP cases, 21% (17/80) were paediatric. Furthermore, 82% (14/17) of those paediatric cases involved upper and lower extremities.

**Table 1:** Demographic of data for each anatomical region of the body.

Anatomical region	FP^1^; *n* (%)	Unsure; *n* (%)	UP^2^	UN^3^	UU^4^
Upper extremity	19 (23.8%)	109 (35.5%)	29	76	4
Shoulder girdle	7 (8.8%)	35 (11.4%)	12	23	0
Chest	8 (10.0%)	29 (9.4%)	1	27	1
Spine	5 (6.3%)	13 (4.2%)	2	11	0
Pelvis	6 (7.5%)	13 (4.2%)	3	10	0
Hip	3 (3.8%)	6 (2.0%)	1	5	0
Lower extremity	32 (40%)	99 (32.2%)	26	70	3
Total	80	307	74	225	8

^1^ False‐positive.

^2^ Unsure‐positive.

^3^ Unsure‐negative.

^4^ Unsure‐unsure.

**Table 2:** Number of errors for each anatomical region of the upper and lower extremities.

Anatomical region	FP^1^	Unsure	UP^2^	UN^3^	UU^4^
Upper extremity	**19**	**109**	**29**	**76**	**4**
Thumb	1	5	2	3	0
Fingers	2	8	2	4	2
Hand	6	22	6	15	1
Wrist	4	24	4	19	1
Forearm	2	16	2	14	0
Elbow	4	30	13	17	0
Humerus	0	4	0	4	0
Lower extremity	**32**	**99**	**26**	**70**	**3**
Toe(s)	1	2	2	0	0
Foot	13	28	8	20	0
Calcaneus	0	2	0	2	0
Ankle	10	37	11	23	3
Tibia‐Fibula	2	5	1	4	0
Knee	6	24	4	20	0
Femur	0	1	0	1	0

^1^ False‐positive.

^2^ Unsure‐positive.

^3^ Unsure‐negative.

^4^ Unsure‐unsure.

The bold numbers in the upper and lower extremity rows represent the sum of the numbers in the column.

**Table 3:** Demographic of paediatric and adult PIE^1^ errors.

	Paediatric; *n* (%)	Adult; *n* (%)	Total
FP^2^	17 (21.2%)	63 (78.8%)	80
Unsure	100 (32.6%)	207 (67.4%)	307
UN^3^	76 (33.8%)	149 (66.2%)	225

^1^ Preliminary image evaluation.

^2^ False‐positive.

^3^ Unsure‐negative.

**Table 4:** Three most common anatomical regions per error type.

Type of error	Anatomical region	Number of errors	Average age (years)	Median age (years)
FP^1^	Foot	13	21.8	16
	Ankle	10	20.9	19
	Chest	8	43.4	38.5
Unsure	Ankle	37	27.0	20
	Shoulder	31	41.6	40
	Elbow	30	27.2	17
UN^2^	Chest	27	49.7	47
	Ankle	23	26.8	16
	Shoulder	21	44.4	45

^1^ False‐positive.

^2^ Unsure‐negative.

Due to the small data set of FP cases, unsure errors were also analysed. Three hundred and seven unsure cases were collected, representing 2.8% of the sample. From the unsure cases, radiologists reported that 24.1% (74/307) of cases were positive for abnormalities. The most common regions that caused unsure errors were the ankle (37/307), shoulder (31/307) and elbow (30/307). In all, 73.2% (225/307) of the unsure cases were negative and 2.6% (8/307) remained inconclusive or unsure. The most common UN cases were the chest (27/307), ankle (23/307) and shoulder (21/307). These are presented in Table 4. Of the 307, 32.6% (100/307) of the unsure cases were paediatric. From the unsure paediatric demographic, 76% (76/100) were negative, 21% (21/100) were positive and 3% (3/100) remained inconclusive after further review from the radiologist (Table 3). Just accounting for extremities, 38.8% (101/260) of FP and unsure errors were found in paediatrics in comparison to adults (Table 5). The three most common UN paediatric cases involved regions of the ankle (12/100), forearm (11/100), elbow and wrist (9/100). Of paediatric FP (^11^) and unsure (100) errors, 88.2% (15/17) and 86.0% (86/100), respectively, occurred in the extremities. Tables 6 and 7 illustrate this further across upper and lower extremities with associated anatomical regions.

**Table 5:** Upper and lower extremity false‐positive and unsure errors in paediatric and adults.

	Paediatric			Adult		
Anatomical region	FP^1^ (*n*)	Unsure (*n*)	Total	FP^1^ (*n*)	Unsure (*n*)	Total
Upper extremity	4	51	55	15	58	73
Lower extremity	11	35	46	22	64	86
			101			159

^1^ False‐positive.

**Table 6:** Upper extremity false‐positive and unsure errors in paediatrics.

Anatomical region	Elbow	Wrist	Forearm	Hand	Finger(s)	Humerus
*n* (%)	15 (27.3)	13 (23.6)	12 (21.8)	11 (20.0)	4 (7.3)	1 (1.8)

**Table 7:** Lower extremity false‐positive and unsure errors in paediatrics.

Anatomical region	Ankle	Foot	Knee	Tibia	Toe(s)
*n* (%)	19 (41.3)	15 (32.6)	8 (17.4)	3 (6.5)	1 (2.2)

## Discussion

This is the largest study to date to report the accuracy of interpreting acute or traumatic injuries when radiographers use PIE in the emergency department. The aims of this study were addressed by investigating which regions of the body caused recorded the most FP errors and explore the underlying mechanisms for these types of errors to further improve the overall accuracy of radiographer PIE. Due to the small sample of FP errors (0.7%) that was collected, unsure errors were also investigated. Recent studies involving the use of PIE in emergency departments has been reported to have an overall accuracy as high as 92%.^12^ Radiologist reporting accuracy has been known to range from 95 to 97%.^13^ Since unsure cases represent 2.8% of this study's results, there is still a learning opportunity that could potentially lift the overall accuracy of the radiographers PIE of 92% to that of the gold standard of a radiologist at 95%.

### False positives

After auditing 11,090 radiographs, 80 FP errors were identified. The most common regions that produced FP errors were the foot, ankle, and chest. Like Alexander‐Bates,^5^ the lower extremities were the most common area of misinterpretation. Although the study discussed FN errors, difficulty in interpreting X‐rays in extremities can be attributed to the fact that these are anatomically dense areas with smaller structures. This can pose limitations on being able to visually identify or assess for pathology, especially when performing a PIE on a suboptimal image viewing monitor. Another point to mention, is that extremities are also highly susceptible to having normal anatomical variants. The foot and ankle are common areas where accessory bones are present which can often mask as pathology, especially when they match the patient's clinical indications.^14^, ^15^ Visualisation of certain pathologies can also extend to pathologies on a chest radiograph, especially rib fractures as it accounted for 75% of the FP chest errors. The limitations that plain X‐ray has in being able to accurately assess for rib fractures has been well documented due to the superimposition and presence of other bony anatomy, lung fields, mediastinum and abdominal viscera.^16^, ^17^ It should be noted that the protocol to assess for rib fractures in the study's department only stipulates a single frontal chest radiograph depending on the patient's presentation, as opposed to including additional rib oblique views. Seeing as additional oblique projections can improve the diagnosis of rib fractures, this may have had an impact on the results.

### Unsure

Unsure data were further subcategorised to focus on UN cases. These cases have a similar nature to FP errors, as upon further review by the radiologist, they were eventually reported as negative. It was found that 73% of the unsure cases were determined as negative in the radiologist report. Not only did this mean that for a large portion of the unsure cases radiographers were unable to identify normal anatomy, but it also meant that radiographers had a higher tendency to categorise cases as ‘unsure’ when there was a suspicion for pathology being present. A raised suspicion could be based off the physical presentation of the patient, clinical indications provided on the imaging request form, or the radiographers interpretation skills. When there is a raised suspicion for pathology, it can be perceived by the radiographer that by interpreting an X‐ray as unsure accompanied by a radiographer PIE, the emergency referrer may find this more beneficial to the patient's care as opposed to ruling out any significant pathologies. Although this may be seen as taking a precaution, there is a possibility that this may contribute to a diagnostic error.

### Unsure negative

The most common UN cases were the chest, ankle and shoulder. As previously mentioned, the nature of chest and ankle radiograph can be inherently difficult to interpret due to the superimposition of overlying organs and bones, presence of anatomical variants or visualisation of structures. On the other hand, a key point of interest was that the age demographic for unsure shoulder cases were much older than other regions of the body. It was discovered that most of the cases presented with degenerative changes such as, joint space narrowing, presence of osteophytes and increased sclerosis. These radiographic signs of degenerative disease can often disguise as acute pathology or even obscure the visualisation of anatomy when making an accurate assessment.^11^

### Paediatric

In Queensland emergency departments, paediatrics account for 21.6% of all patient presentations.^18^ Overall, the paediatric population accounted for 21.3% of the FP cases and 33.6% of all the unsure cases. It is important to note that for 76% of the unsure paediatric cases, radiographers were unable to recognise normal variants in anatomy. Among both types of errors, the ankle, elbow and foot were regions that caused the most misinterpretation errors. These regions also coincide with errors made by radiologists and emergency doctors in previous studies that assessed the interpretation of paediatric X‐rays.^19^ Unsurprisingly, due to the developmental and complex nature of the paediatric anatomy, being able to distinguish between subtle fractures from growth plates and ossification sites from avulsion fractures are difficult tasks for all practitioners and have been proven to lead to incorrect or inconclusive X‐ray interpretation.

## Study Strengths and Limitations

The greatest strength of this study was the large size and long study period of which data was collected. To date, this is the largest study to assess the accuracy of radiographer PIE's. Being able to analyse 11,090 cases over a period of 5 years gives the results greater validity. Another strength of this study was that through retrospective analysis, we were able to obtain data regarding patient age. Data regarding patient age in relation to PIE has also yet to be investigated, thus improving the quality and depth of findings. Limitations to this study was that radiographers were inconsistent with documenting their reasons for categorising a case as unsure. Without this information, it was hard to discern what structures or features of a particular X‐ray made it particularly difficult to interpret. Thus, only the frequency of errors made per anatomical region could be analysed as opposed to more critical details such as image quality, presence of artefact or anatomical appearance. A future study that collects and analyses this additional detail would be of interest to the medical imaging profession. Furthermore, while the inclusion of unsure cases provide additional insight to the study, it does introduce bias into the analysis. A FP error indicates that a radiographer has the confidence to document a PIE. Conversely, unsure cases occur when the radiographer lacks the confidence to make a definitive PIE. While FP and unsure cases are categorised as errors, the difference in the radiographer's confidence between the two instances needs to be taken into consideration when interpreting the data.

## Conclusion

This is the largest study to report specific types of injuries and interpretation errors when radiographers perform PIE. The audit reviewed 11,090 images over a 5‐year period, demonstrating an overall accuracy of 87.7%. This study uncovered that the foot, ankle and chest regions caused the most FP errors, while ankle, shoulder and elbow caused the most unsure cases. Additionally, a large proportion of FP and unsure cases were paediatric. Due to the developmental and complex nature of paediatric anatomy, interpreting paediatric X‐rays was shown to be a weakness for radiographers. Findings from this study should be used to tailor education specific to radiographer image interpretation. Improving radiographers' image interpretation skills can assist in improving a referrer's diagnostic accuracy, thus improving patient outcomes.

## Conflict of Interest

The authors declare no conflict of interest.

## Data Availability

The data that support the findings of this study are available from the corresponding author upon reasonable request.
